# *Cryptopimpla* (Hymenoptera, Ichneumonidae, Banchinae) of South Korea, with description of two new species

**DOI:** 10.3897/zookeys.830.31974

**Published:** 2019-03-14

**Authors:** Gyu-Won Kang, Janko Kolarov, Jong-Wook Lee

**Affiliations:** 1 Department of Life Sciences, Yeungnam University, Gyeongsan, South Korea Yeungnam University Gyeongsan South Korea; 2 Faculty of Pedagogy, University of Plovdiv, 24 Tsar Assen Str., 4000 Plovdiv, Bulgaria University of Plovdiv Plovdiv Bulgaria

**Keywords:** Atrophini, Eastern Palearctic, ichneumon wasp, species description, new records, taxonomy

## Abstract

The genus *Cryptopimpla* Taschenberg is recorded for the first time in South Korea. Four species are recognized; among these, two species, *C.aspeculosus* Kang & Lee, **sp. n.** and *C.pentagonalis* Kang & Lee, **sp. n.**, are described as new to science. For the other two species, *C.brevigena* Kuslitzkii and *C.carinifacialis* Sheng, the males were hitherto unknown and are described here. An illustrated identification key is provided for the species of *Cryptopimpla* known from South Korea.

## Introduction

*Cryptopimpla* Taschenberg is a moderately large genus with a worldwide distribution, containing 57 species (Reynolds Berry and van Noort 2016; Yu et al. 2016). Among these, 15 species are from the Eastern Palearctic region. Additionally, the Oriental, and Western Palearctic regions contain 15 species each, only one from the Neotropical region, and eight from the Nearctic region (Yu et al. 2016). Recently, Sheng (2011) described five new species from China. As a member of the tribe Atrophini, the genus can be distinguished by the following combination of traits: occipital carina joining hypostomal carina; dorsal tooth of mandible longer than ventral tooth; epomia absent; ventral half of mesopleuron weakly convex; forewing with areolet; hindwing vein 1/cu slightly longer than cu-a; ovipositor sheath 0.5–1.0 times as long as hind tibia; ovipositor tip with subapical dorsal notch (Townes 1970). *Cryptopimpla* species are parasitoids of leaf-rolling larvae of Lepidoptera (Yu et al. 2016). Unfortunately, the hosts from the species of *Cryptopimpla* that occur in South Korea remain unknown. This work aims to provide a taxonomic account of the *Cryptopimpla* from South Korea, recording the genus for the first time, and describing two new species to sciences.

## Materials and methods

Specimens were collected by sweeping and Malaise traps, and are deposited in the animal systematic laboratory of Yeungnam University (Gyeongsan, South Korea). Morphological terminology follows that of the American Entomological Institute website (http://www.amentinst.org/GIN/morphology.php), wing vein nomenclature is based on Ross (1936). Specimens were examined using an AxioCam MRc5 camera attached to a stereo microscope (Zeiss SteREO Discovery V20; Carl Zeiss, Göttingen, Germany), processed using AxioVision SE64 software (Carl Zeiss), and optimized with a Delta imaging system (i-solution, IMT i-Solution Inc. Vancouver, Canada).

Abbreviations used in this paper are as follows: **CN**, Chungcheongnam-do; **GB**, Gyeongsangbuk-do; **GG**, Gyeonggi-do; **GW**, Gangwon-do; **JB**, Jeollabuk-do; **JN**, Jeollanam-do; **TL**, Type Locality and **TD**, Type depository. Abbreviations for collections are as follows: **MF**, Ministry of Forestry, General Station of Forestry Pest and Management, Shenyang, Lioning, China; **YNU**, Laboratory of Animal Systematics and Taxonomy, Department of Life Sciences, Yeungnam University, Gyeongsan, South Korea and **ZI**: Zoological Institute, Academy of Sciences, St. Petersburg, Russia.

## Results

### Systematics

#### Genus Cryptopimpla

Taxon classificationAnimaliaHymenopteraIchneumonidae

Taschenberg, 1863

Cryptopimpla Taschenberg, 1863: 292. Type species: Phytodietusblandus Gravenhorst, 1829
Aphanodon Förster, 1869: 166. Type species: Phytodietuserrabundus Gravenhorst, 1829
Xenacis Förster, 1869: 167. Type species: Lissonotacaligata Gravenhorst, 1829
Xenocornia Schmiedeknecht, 1900: 334. Type species: Xenocorniasolitaria Schmiedeknech, 1900
Harrimaniella Ashmead, 1900: 52. Type species: Harrimaniellayukakensis Ashmead, 1900

### Key to species of the genus *Cryptopimpla* from South Korea

**Table:** 

1	Malar space shorter than 0.3 times basal width of mandible. Tarsal claws simple. Propodeum without pleural and posterior transverse carina (Fig. 2C)	*** C. brevigena ***
–	Malar space longer than 0.3 times basal width of mandible. Tarsal claws simple or pectinate. Propodeum with pleural and posterior transverse carina (Fig. 4C)	**2**
2	3rs-m vein present only basal, vestigial (Fig. 1E). Antenna with less than 40 flagellomeres	***C.aspeculosus* sp. n.**
–	3rs-m vein complete (Fig. 3E). Antenna with more than 40 flagellomeres	**3**
3	Areolet narrowly petiolate (Fig. 3E). Face with strong median carina on dorsal half. Tergites with yellow apical band (Fig. 3A)	*** C. carinifacialis ***
–	Areolet pentagonal (Fig. 4E). Face without strong median carina on dorsal half. Tergites without yellow apical band (Fig. 4A)	***C.pentagonalis* sp. n.**

#### Cryptopimpla aspeculosus

Taxon classificationAnimaliaHymenopteraIchneumonidae

Kang & Lee sp. n.

http://zoobank.org/08018962-545B-40D6-93D6-3F1D13D68D97

Fig. 1

##### Male.

**Figure 1.**
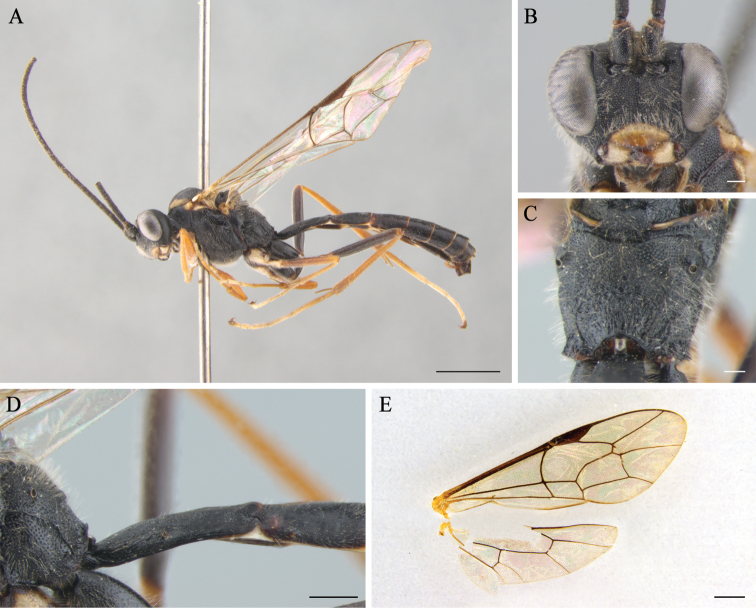
*Cryptopimplaaspeculosus* sp. n. (holotype, male) **A** habitus in lateral view **B** head in frontal view **C** propodeum **D** first tergite in lateral view **E** wings. Scale bars: 2.0 mm (**A**); 0.2 mm (**B, C**); 0.5 mm (**D**); 1.0 mm (**E**).

Forewing 7.6 mm (7.6–7.7 mm, n = 2), body 10.3 mm (10.3–10.5 mm, n = 2) long (Fig. 1A).

##### Head.

In dorsal view, 2.3 times as wide as long, and distinctly narrowed behind, densely and coarsely punctate with coriaceous between punctures. Diameter of median ocellus 0.6 times as long as distance between lateral ocellus and compound eye. Flagellum with 38 elongated flagellomeres; 1^st^ flagellomere 3.5 times as long as wide. Occipital carina narrowly curved from above, reaching hypostomal carina above the base of mandible. Face weakly convex medially, 1.7 times as wide as long, densely and rather coarsely punctate, without carina between antennal sockets (Fig. 1B). Clypeus weakly convex; 2.3 times as wide as long, with sparse punctures and blunt fore ridge. Malar space 0.7 times as long as basal width of mandible.

##### Mesosoma.

Coarsely and densely punctate on the coriaceous surface; 1.6 times as long as high. Notaulus long and shallow. Epicnemial carina reaches near the ventral hind margin of pronotum, but does not join it. Propodeum slightly straight in lateral view, with posterior transverse, pleural carinae and weak median longitudinal carina; propodeal spiracle moderately large, oval (Fig. 1C). Legs very slender; hind femur 7.1 times as long as wide; hind inner tibial spur 0.42 times as long as 1^st^ tarsal segment; ratio of hind tarsal segments are 5.2:2.3:1.5:1.0:1.3; all tarsal claws simple. Forewing with incomplete 3rs-m; 2m-cu with a single bulla; 1cu-a vein weakly postfurcal; vein 2-Cu as long as 2cu-a. Hindwing with 8 distal hamuli; vein 1/cu about 1.5 times as long as cu-a (Fig. 1E).

##### Metasoma.

1^st^ tergite 1.7 times as long as wide, with prominent spiracle at basal 0.45 (Fig. 1D). 2^nd^ tergite square. All tergites finely coriaceous. 4^th^ and apical third of 3^rd^ tergite with sparse punctures.

##### Color.

Body black; basal half of clypeus and mandible, palpi, collar, hind ventral and dorsal angle of pronotum, wide lateral stripe on mesonotum from tegula to mid lobe, tegula, subtegular ridge, fore and mid coxa, fore trochanter from below and hind tarsus, except basal 3/4 of basitarsus and apical half of last tarsal segment yellow; fore and mid femora, tibiae and tarsi reddish, hind tibia and hind basitarsus (except apical 1/4) red; apical half of last tarsal segment of hind leg dark brown.

##### Female.

Unknown.

##### Specimens examined: Holotype.

male, South Korea, Icheon, Mt. Seolbongsan, 1 April 1984, Y.S. Kim (YNU);

##### Paratype.

1 male, South Korea, GG, Namyangju-si, Bogwangsa, 13 April 1984, J.W. Lee (YNU).

##### Distribution.

South Korea (new record).

##### Etymology.

The name comes from Latin “speculo”, *aspeculosus* meaning “without areolet”.

##### Remarks.

The species is similar to *C.brevigena*, from which it differs by the presence of pleural and posterior transverse carinae and the entirely black face. Furthermore, the malar space in *C.brevigena* is 0.3 times as long as the basal width of the mandible while in *C.aspeculosus* it is 0.7 times. Additionally, this species is easily separated from other two species (*C.carinifacialis* and *C.pentagonalis*) as follows: the 3rs-m vein in *C.aspeculosus* is only present in the basal part, while in the other two species have a complete 3rs-m vein.

#### Cryptopimpla brevigena

Taxon classificationAnimaliaHymenopteraIchneumonidae

Kuslitzkii, 2007

Fig. 2

Cryptopimpla brevigena Kuslitzkii, 2007: 453. Type: ♀, TL: Russia – Primorsky Kray; TD: ZI.

##### Male.

**Figure 2.**
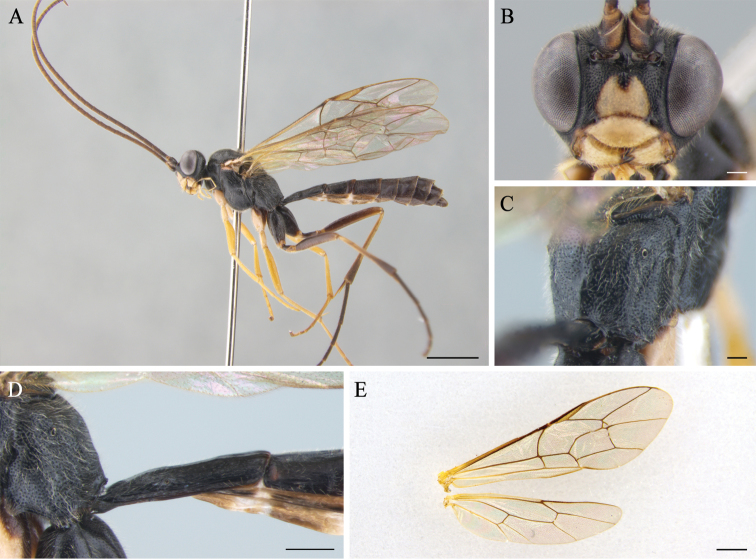
*Cryptopimplabrevigena* (male) **A** habitus in lateral view **B** head in frontal view **C** propodeum **D** first tergite in lateral view **E** wings. Scale bars: 2.0 mm (**A**); 0.2 mm (**B**, **C**); 0.5 mm (**D**); 1.0 mm (**E**).

Forewing 7.7 mm (7.4–8.0 mm, n = 42), body 9.5 mm (9.3–9.7 mm, n = 42) long (Fig. 2A).

##### Head.

In dorsal view, 2.0 times as wide as long, narrowed behind, round, densely punctate with coriaceous between punctures. Diameter of median ocellus 0.64 times as long as distance between compound eye and lateral ocellus. Flagellum with 46 (43–48, n = 40) elongated flagellomeres; 1^st^ flagellomere 3.8 times as long as wide. Occipital carina evenly curved from above, meeting hypostomal carina near base of mandible. Face convex medially, 1.7 times as wide as long, densely and coarsely punctate (Fig. 2B). Clypeus strongly convex; 2.5 times as wide as long, with more sparse punctures and blunt fore ridge. Malar space 0.28 times as long as basal width of mandible.

##### Mesosoma.

Moderately coarsely and densely punctate, 1.5 times as long as high. Notaulus short and very shallow. Epicnemial carina ends near middle of epicnemium and does not reach frontal ridge of mesopleuron. Propodeum convex in lateral view, without posterior transverse and pleural carinae (Fig. 2C); propodeal spiracle moderately small, oval. Legs slender; hind femur 6.5 times as long as wide; hind inner tibial spur 0.5 times as long as basitarsus; ratio of hind tarsal segments 4.4:2.2:1.6:1.0:1.1; all tarsal claws simple. Areolet petiolate; 2m-cu with two bullae; 1cu-a vein weakly postfurcal; vein 2-Cu slightly longer than 2cu-a. Hindwing with 9 distal hamuli; vein 1/cu about 1.6 times as long as cu-a, reclivous (Fig. 2E).

##### Metasoma.

1^st^ tergite 2.2 times as long as wide, with spiracles before its middle (Fig. 2D); lateral carina developed on entire length of tergite. 2^nd^ tergite 1.4 times as long as its apical width. All tergites coriaceous with fine punctures.

##### Color.

Body black. Scape and pedicel from below, a large triangular spot on face centrum, clypeus entirely, mandible except teeth, palpi, hind dorsal corner of pronotum, tegula, subtegular ridge, fore and mid coxa and trochanters, and hind trochanters from below yellow. Fore and mid legs orange yellow, basal hind tibia faint whitish.

##### Female.

Flagellum with 40–44 segments (n=17). Hindwing with 7 distal hamuli.

##### Specimens examined.

South Korea: 1 male, CN, Seosan-si, Haemi-myeon, Daegok-ri, Hanseo Univ., 30 April–9 May 2006; 1 male, CN, Taean-gun, Geunheung-myeon, 18 July 1994, M.J. Shin; 1 female, GB, Uiseong-gun, Danchon-myeon, Sanghwa-ri, 7 May 2016, J.W. Lee; 1 male, GB, Ulleung-gun, Buk-myeon, Naribunji, 27 May 2016, G.H. Ko; 1 male, GB, Yeongju-si, Punggi-eup, Jungnyeong, 12–22 June 2009; 1 female, ditto, 22 June–3 July 2009, C.J. Kim; 1 female, GG, Geumgok, 11 June 1983, H.I. J.; 1 male, GG, Namyangju-si, Choan-myeon, Songchon-ri, Mt. Ungilsan, 27 May–10 June 2009, J.O. Lim; 1 female, GG, Namyangju-si, Sudong-myeon, Mt. Chungnyeongsan, 12 July 1980, J.I. Kim; 1 female, GG, Namyangju-si, Mt. Cheonmasan, 18 June 1983, J.W. Lee; 1 female, GW, Goheung-gun, Yeongam-myeon, Paryeong-ro, Geumsa-ri, Mt. Paryeongsan Forest Resort, 13 April 2012; 3 females, GW, Pyeongchang-gun, Yongpyeong-myeon, Mt. Gyebangsan, 28 June–12 August 2012, J.Y. Park; 1 female, GW, Wonju-si Maeji-ri Yonsei Univ. Campus, 1 August 1999, S.W. Kim, D.W. Kim, and D.Y. Kim; 3 females and 31 males, GW, Wonju-si, Panbu-myeon, Mt. Baegunsan, 25 May–26 July 2012, H.Y. Han; 1 female, JB, Jangsu-gun, Jangsu-eup, Daeseong-ri, San 258-1, Mt. Palgongsan, 17 June–2 July 2015, J.W. Lee; 4 males, JB, Namwon-si, Sanneae-myeon, Dalgung valley (M.T.), 1–9 July 2001, J.W. Lee; 1 female and 1 male, JN, Gwangyang-si, Daap-myeon, Hacheon-ri, San123, Mt. Baegunsan, 17 June–2 July 2015, J.W. Lee; 1 female, Seoul, Gwanak-gu, Mt. Gwanaksan, 19 June 1983, J.H. Han; 1 female, Seoul, Nowon-gu, Mt. Suraksan, 25 May 1997, H.C. Lim; 1 male, Seoul, Seocho-gu, Wonji-dong, Mt. Cheonggyesan, 4 June 1989, J.W. Lee; 1 female, ditto, 17 June 1998, Y.G. Park.

##### Distribution.

South Korea (new record), Russia (Primorsky Kray, Sakhalin Oblast)

##### Remarks.

This species is recorded for the first time from South Korea. It is easily distinguished from other South Korean species by having simple tarsal claws and the absence of posterior transverse and pleural carinae. The male is newly described from South Korea in this study.

#### Cryptopimpla carinifacialis

Taxon classificationAnimaliaHymenopteraIchneumonidae

Sheng, 2011

Fig. 3

Cryptopimpla carinifacialis Sheng, 2011: 32. Type: ♀, TL: China-Jiangxi; TD: MF.

##### Male.

**Figure 3.**
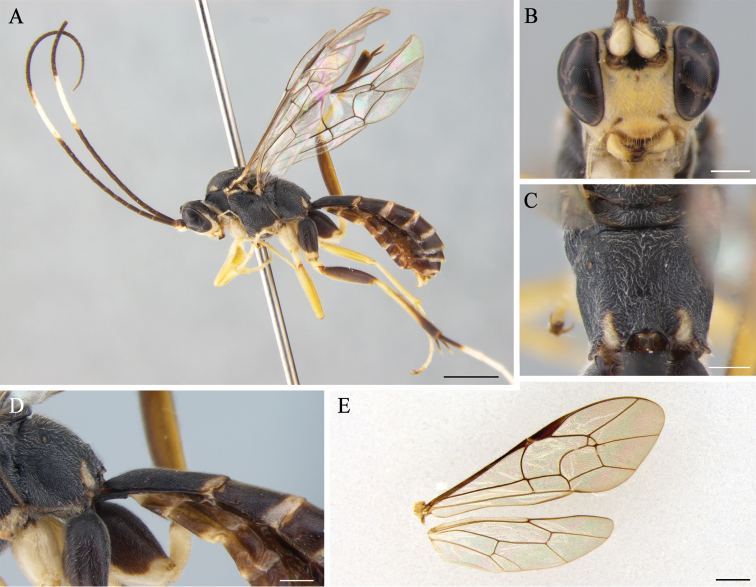
*Cryptopimplacarinifacialis* (male) **A** habitus in lateral view **B** head in frontal view **C** propodeum **D** first tergite in lateral view **E** wings. Scale bars: 2.0 mm (**A**); 0.5 mm (**B,C, D**); 1.0 mm (**E**).

Forewing 7.8 mm, body 9.3 mm long (Fig. 3A).

##### Head.

In dorsal view, 2.0 times as wide as long, narrowed behind roundly, densely and finely punctate, coriaceous between punctures. Diameter of median ocellus 0.9 times as long as distance between lateral ocellus and compound eye. Flagellum with 49 elongated segments; 1^st^ flagellomere 4.0 times as long as wide. Occipital carina complete entirely, reaching hypostomal carina above base of mandible. Inner profile of basal half of flagellum without a strong longitudinal carina (present only in female). Face convex medially, 1.3 times as wide as long, coriaceous and densely punctate, with short, distinct median longitudinal carina just between and below antennal sockets (Fig. 3B). Clypeus 2.4 times as wide as long; sparsely punctured at basal half. Malar space 0.5 times as long as basal width of mandible.

##### Mesosoma.

Densely punctate on the coriaceous surface, 1.5 times as long as high. Notaulus short and very shallow. Epicnemial carina reaches near the ventral hind, not reaching frontal ridge of mesopleuron. Propodeum slightly straight in lateral view, posterior transverse carina weak, more evident in the middle (Fig. 3C); propodeal spiracle moderately large, oval. Legs moderately slender, hind femur 5.6 times as long as wide. Ratio of hind tarsal segments 4.8:2.3:1.6:1.0:1.2. Tarsal claws pectinate. Forewing with complete areolet narrowly petiolate; 2m-cu connects areolet in its outer angle, with two bullae; 1cu-a vein weakly postfurcal; vein 2-Cu slightly longer than 2cu-a. Hindwing with 10 distal hamuli; vein 1/cu about 3.0 times as long as cu-a, weakly reclival (Fig. 3E).

##### Metasoma.

1^st^ tergite 2.0 times as long as wide, without median dorsal and lateral carinae; spiracle prominent laterally, situated well before mid tergite (Fig. 3D). Anterior three tergites punctured on coriaceous surface, matt; following tergites with fine punctures and shiny.

##### Color.

Body black, with numerous yellow marks; scape and pedicel from below, 11^th^ to 18^th^ flagellomeres, facial orbit, face, clypeus, mandible, malar space, ventral 2/3 of outer eye orbit, palpi, fore margin of pronotum widely and hind dorsal angle, two antero-lateral spots and two spots on mid mesonotum, scutellum except basally and apically, subtegular ridge, a spot on hind low ventral part of mesopleuron above mid coxa to near epicnemial carina, two spots on propodeum apically, fore and mid legs, all trochanters, hind coxa apically, basal half of hind tibia, hind tarsal segments except basal half of 1^st^ one, all tergites apically yellow; hind femur darkened from above, red-yellow from below.

##### Female.

Flagellum with 46 segments. Hindwing with 9 distal hamuli. There is some variation in coloration from the original description: scape and pedicel entirely black; inner eye orbit and ventral half of outer orbit, apical half of clypeus, mandible except basally and teeth yellow; meso – and metapleuron entirely black, but propodeum with two yellow spots apically; apical part of hind basitarsus, and 2^nd^ to 4^th^ tarsal segments entirely yellow; 1^st^ and 2^nd^ tergites with two latero-apical yellow spots.

##### Specimens examined.

South Korea: 1 male, GG, Gapyeong-gun, Sangtan-ri, 14 June 1992, Y.H. Baek; 1 female, GW, Samcheok-si, Hajang-myeon, Mt. Jungbongsan, 19 October 2008, H.S. Lee.

##### Distribution.

South Korea (new record), China (Jiangxi).

##### Remarks.

Only the female of the species has been described to date (Sheng 2011; Sheng et al. 2013). The male is newly described in this study.

#### Cryptopimpla pentagonalis

Taxon classificationAnimaliaHymenopteraIchneumonidae

Kang & Lee sp. n.

http://zoobank.org/68DA02AF-17A7-4DAD-8C5D-112C57EDB8B3

Fig. 4

##### Female.

**Figure 4.**
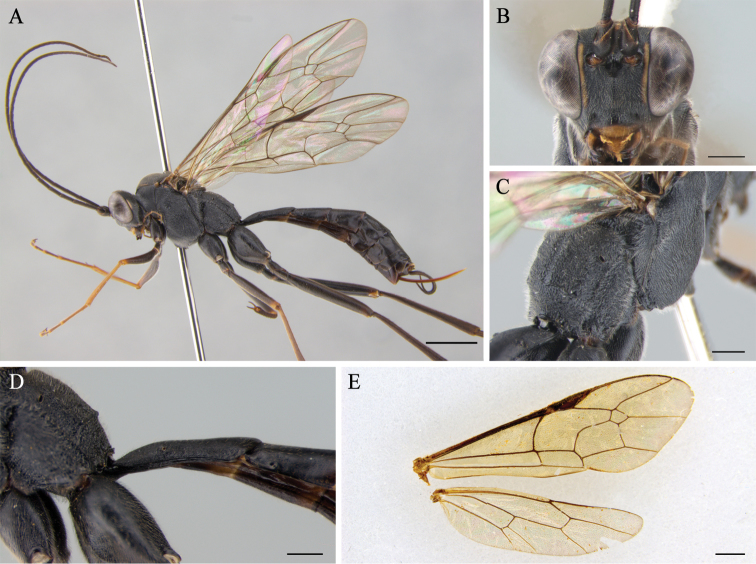
*Cryptopimplapentagonalis* sp. n. (female) **A** habitus in lateral view (holotype) **B** head in frontal view (holotype) **C** propodeum (holotype) **D** first tergite in lateral view (holotype) **E** wings (paratype). Scale bars: 2.0 mm (**A**); 0.5 mm (**B, C, D**); 1.0 mm (**E**).

Forewing 9.3 mm (8.8–9.3 mm, n = 7), body 9.8 mm (9.5–9.8 mm, n = 7), ovipositor sheath 2.6 mm (2.4–2.6 mm, n = 7) long (Fig. 4A).

##### Head.

In dorsal view, 3.4 times as wide as long, narrowed behind distinctly, densely punctate between punctures. Diameter of median ocellus 0.77 times as long as distance between lateral ocellus and compound eye. Flagellum with 42 (in paratypes 41–43, n = 6) elongated segments; 1^st^ flagellomere 5.0 times as long as wide. Occipital carina weakly curved from above, reaching hypostomal carina at base of mandible. Face slightly convex medially, 1.4 times as wide as long, densely and rather coarsely punctate (Fig. 4) without carina between antennal sockets (Fig. 4B). Clypeus convex; 1.8 times as wide as long with sparse punctures and blunt fore ridge. Malar space 0.6 times as long as basal width of mandible.

##### Mesosoma.

Densely and coarsely punctate on the coriaceous surface, 1.6 times as long as high. Notaulus short and very weak. Epicnemial carina reaching near ventral hind margin of pronotum. Propodeum convex in lateral view, with only posterior transverse and pleural carinae; spiracle moderately large, oval (Fig. 4C). Legs slender; hind femur 6.5 times as long as wide; hind inner tibial spur 0.42 times as long as basitarsus; ratio of hind tarsal segments 6.0:2.7:1.8:1.0:1.3; tarsal claws fully pectinate. Forewing with pentagonal areolet; vein 3rs-m distinctly present; 2m-cu with two bullae; 1cu-a vein weakly postfurcal; vein 2-Cu slightly longer than 2cu-a. Hindwing with 9 distal hamuli; vein 1/cu about 2.0 times as long as cu-a, reclivous (Fig. 4E).

##### Metasoma.

1^st^ tergite 2.5 times as long as wide apically, without median and lateral carinae; spiracle situated before middle (Fig. 4D). 2^nd^ tergite 1.0 times as long as apical width. Ovipositor sheath 0.63 times as long as hind tibia. Ovipositor straight and compressed, with subapical dorsal notch.

##### Color.

Black. Inner face of orbit, ventral part of frontal orbit, spot on top of eye orbit opposite to lateral ocellus, facial orbit, fore ridge of pronotum medially, thin lateral stripe on antero-lateral portion of mesonotum and 2^nd^ to 4^th^ tarsal segments of hind leg yellow. Apical half of clypeus, fore tibia, and tarsus reddish-brown.

##### Male.

Unknown.

##### Specimens examined. Holotype:

South Korea: female, GW, Jeongseon-gun, Gohan-eup, Haiwongil, Mountain condo, 9 July 2010 (YNU).

##### Paratypes.

2 females, GW, Jeongseon-gun, Gohan-eup, Haiwongil, Mountain condo, 9 July 2010 (YNU); 3 females, GW, Jeongseon-gun, Gohan-eup, Mt. Baegunsan, 9 July 2010 (YNU); 1 female, GW, Wonju-si, Panbu-myeon, Mt. Baegunsan, 25 May–26 July 2012, H.Y. Han (YNU).

##### Distribution.

South Korea (new record).

##### Etymology.

The name comes from Latin “Pentagonum”, *pentagonalis* means “pentagon areola”.

##### Remarks.

With the pentagonal areolet, black metasoma, hind coxa and femur, this species is similar to *Cryptopimplahenanensis* Sheng, but differs in the propodeum structure and body coloration; in *C.pentagonalis* the mesosoma is entirely black while in *C.henanensis* it has more yellow spots. The latter species also has more flagellomeres; in *C.henanensis* with 46 segments while in *C.pentagonalis* with an average of 42 segments.

## Supplementary Material

XML Treatment for Cryptopimpla

XML Treatment for Cryptopimpla aspeculosus

XML Treatment for Cryptopimpla brevigena

XML Treatment for Cryptopimpla carinifacialis

XML Treatment for Cryptopimpla pentagonalis
